# Restless Legs Syndrome in Chronic Kidney Disease- a Systematic Review

**DOI:** 10.5334/tohm.752

**Published:** 2023-03-29

**Authors:** Yasaman Safarpour, Nosratola D. Vaziri, Bahman Jabbari

**Affiliations:** 1 Department of Psychiatry, Sleep Medicine Division, Stanford Health Care, US; 2 Division of Nephrology and Hypertension, Departments of Medicine, Physiology and Biophysics, University of California, Irvine, Irvine, California, US; 3 Department of Neurology, Yale University School of Medicine, US

**Keywords:** Restless legs syndrome, Movement disorders, Renal failure, Hemodialysis, Sleep Disorders

## Abstract

**Objectives:**

The objective of this review is to provide updated information on the epidemiology, correlating factors and treatment of chronic kidney disease associated restless legs syndrome (CKD-A-RLS) in both adult and pediatric population.

**Materials and Methods:**

We have reviewed the Medline search and Google Scholar search up to May 2022, using key words restless legs syndrome, chronic kidney disease and hemodialysis and kidney transplant. The reviewed articles were studied for epidemiology, correlating factors, as well as pharmacologic and non-pharmacologic treatment options.

**Results:**

Our search revealed 175 articles, 111 were clinical trials or cross- sectional studies and 64 were review articles. All 111 articles were retrieved and studied in detail. Of these, 105 focused on adults and 6 on children. A majority of studies on dialysis patients reported a prevalence between 15–30%, which is notably higher than prevalence of RLS in general population (5–10%). The correlation between presence of CKD-A-RLS with age, gender, abnormalities of hemogram, iron, ferritin, serum lipids, electrolytes and parathyroid hormones were also reviewed. The results were inconsistent and controversial. Limited studies have reported on the treatment of CKD-A-RLS. Non-pharmacological treatment focused on the effect(s) of exercise, acupuncture, massage with different oils and infra-red light whereas, pharmacologic treatment options include the effects of dopaminergic drugs, Alpha2-Delta ligands (gabapentin and pregabalin), vitamins E and C, and intravenous iron infusion.

**Conclusion:**

This updated review showed that RLS is two to three times more common in patients with CKD compared to the general population. More patients with CKD-A-RLS demonstrated increased mortality, increased incidence of cardiovascular accident, depression, insomnia and impaired quality of life than those with CKD without RLS. Dopaminergic drugs such as levodopa, ropinirole, pramipexole and rotigotine as well as calcium channel blockers (gabapentin and pregabalin) are helpful for treatment of RLS. High quality studies with these agents are currently underway and hopefully confirm the efficacy and practicality of using these drugs in CKD-A-RLS. Some studies have shown that aerobic exercise and massage with lavender oil can improve symptoms of CKD-A- RLS suggesting that these measures can be useful as adjunct therapy.

## Introduction

The definition and classification of chronic kidney disease (CKD) have evolved over time. The current international guidelines define this condition as decreased kidney function characterized by a glomerular filtration rate (GFR) of less than 60 ml/min per 1·73 m², or markers of kidney damage, or both, of at least 3 months duration, regardless of the underlying cause. CKD is very prevalent in the general adult population. Data from the United States estimate a prevalence of 13.1% among adults, which has increased over time. The burden of CKD is substantial. According to WHO global health estimates, 864 226 deaths (or 1·5% of deaths worldwide) were attributable to this condition in 2012. Ranked fourteenth in the list of leading causes of death, CKD accounted for 12·2 deaths per 100,000 people. Projections from the Global Health Observatory suggest that the death rate from CKD will continue to increase to reach 14 per 100,000 people by 2030. CKD is also associated with substantial morbidity. Worldwide, CKD accounted for 2,968,600 (1·1%) of disability-adjusted life-years and 2,546,700 (1·3%) of life-years lost in 2012. Patients with CKD require monitoring for complications such as metabolic abnormalities, anemia, CKD associated mineral bone disease, and cardiovascular diseases [1,2].

Patients with chronic kidney disease are commonly affected with various types of sleep disorders. Sleep disorders have been associated with increased cardiovascular risk and may contribute to the morbidity and mortality of people with advanced (stages 4 to 5) CKD and those treated with dialysis [3]. Within the spectrum of sleep disorders, restless legs syndrome (RLS) causes a disturbance in sleep through an irresistible desire to move one’s legs. Symptoms of RLS are more common in patients with CKD than in the general population [4,5].

Restless legs syndrome, also known as Willis-Ekbom disease (WED), is a sensorimotor disorder characterized by an irresistible urge to move the legs. The urge is usually accompanied by an uncomfortable sensation in the legs that occurs in the evening or night and is partially or totally relieved by movement [6]. Brain iron deficiency and dopaminergic neurotransmission abnormalities play a central role in the pathogenesis of RLS, along with other nondopaminergic systems, although the exact mechanisms are still unclear. The cause of most cases of RLS is unknown, and hence is called primary (idiopathic) RLS. Secondary RLS occurs in association with a variety of systemic disorders especially iron deficiency and chronic renal insufficiency [7].

The initial management approach to essential RLS should include measuring serum ferritin and transferrin-percent saturation, with iron-replacement therapy indicated when these measures are below the low-to-normal range. There is limited evidence of nonpharmacologic treatment in primary RLS. In moderate to severe RLS, pharmacologic treatment may be considered. There is strong evidence for efficacy of both Alpha2-Delta Ligands (gabapentin and pregabalin) and dopamine agonists in the therapy for RLS. Unfortunately, a growing body of evidence over the last decade has indicated disturbing side effects associated with dopaminergic therapies. Most significantly, a large proportion of RLS patients treated with dopaminergic drugs (direct agonists such as ropinirole) develop augmentation syndrome. Augmentation is characterized by earlier appearance of the symptoms during the day, often associated with more intensity. Prevalence rates for dopamine agonist–related augmentation vary from less than 10% in the short term to 42% to 68% after approximately 10 years of treatment. In addition, excessive daytime sleepiness with sleep attacks particularly in patients with comorbid parkinsonism, impulse control disorder symptoms, as well as dose related adverse effects, such as dizziness and drowsiness may develop in patients on dopamine agonist medications. Second-line therapies include intravenous iron infusion in those who are intolerant of oral iron intake and/or those having augmentation with intense, severe RLS symptoms, and opioids including tramadol, oxycodone, and methadone [8,9,10].

There is evidence that chronic RLS makes the patients prone to cardiac and cerebrovascular accidents although there is a need for more careful studies in this area [11].

## Research Design

A Medline search and Google Scholar search was conducted up to May 1^st^, 2022, crossing the term restless legs syndrome and chronic kidney disease (CKD) and additionally with hemodialysis. The search included only articles published in English language. The number of relevant articles in adult and pediatric literature were presented in a Prisma monograph. Prevalence of RLS was investigated in CKD patients on dialysis and off dialysis as well in hemodialysis versus peritoneal dialysis and after kidney transplantation.

The search included data on presence or lack of correlation between CKD-associated- RLS (CKD-A-RLS) and gender, age, basic metabolic index, serum albumin, serum lipid profile and presence of comorbidities (such as diabetes and hypertension). Additionally, correlations were searched and recorded between CKD-A-RLS and a large number of metabolic and hormonal factors including serum electrolytes (in particular calcium and phosphorus), serum iron, hemoglobin, ferritin, transferrin saturation, parathyroid hormone level as well as stages of kidney dysfunction and markers of kidney dysfunction (glomerular filtration rate, serum creatinine and BUN). The search also included data supporting or refuting correlation between CKD-A-RLS with dialysis parameters such as frequency and duration of dialysis as well as type of dialysate used for treatment of renal failure. The issue of mortality in CKD-A-RLS syndrome was searched for and analyzed.

Data from blinded and open label studies on treatment of CKD-A-RLS were compiled and analyzed. The search included data from commonly used agents such as dopaminergic and antiepileptic drugs and certain opioids as well as newer and experimental drugs. Information from case reports was excluded. Important treatment protocols in progress were mentioned and briefly discussed.

The data from non- pharmacological clinical trials such as those related to the use of different exercise modalities, acupuncture and herbal treatments were noted and recorded. Fischer exact test was used for to detect statistical significance between small data values.

## Results

**Figure 1:**
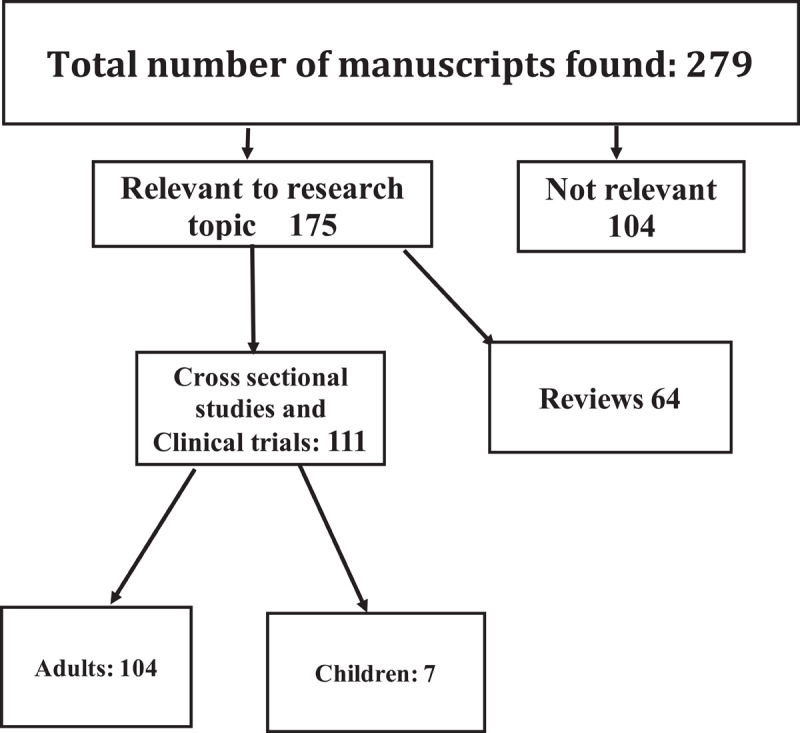
Research Prisma.

The search, performed up to May^1st^ 2022, disclosed 510 articles. Two authors reviewed the literature under this subject independently. After exclusion of duplications (231 duplications), 279 articles remained (Figure 1- Prisma). Of these, 104 articles were excluded (not relevant to the topic, in languages other than English, case reports). Of the remaining 175 articles, 111 were clinical trials or cross- sectional studies and 64 were review articles. All 111 articles were retrieved and studied in detail. Of these, 105 focused on adults and 6 on children.

The reported prevalence of CKD-A-RLS varied among different studies. Majority of studies on dialysis patients reported a prevalence between 15–30%, notably higher than prevalence of RLS in general population (5–10%) [12]. Seven studies described much higher values, ranging from 51–84% [13,14,15,16,17,18,19]. One study also reported a high prevalence of CKD-A-RLS (37.1%) in non-dialyzed patients [20]. One study reported a very low incidence of CKD-A-RLS in Asian Indian population of only 1.5% (zero in controls) [21].

Seven studies reported on the prevalence of CKD-A-RLS after kidney transplant. In 6 of these studies, the prevalence value was considerably lower than that reported among dialysis patients and corresponded with RLS prevalence in general population [22,23,24,25,26]. six studies reported increased mortality in patients affected by CKD-A-RLS [26,27,28,29,30,31], whereas two studies found no significant change in mortality in the affected patients [32,33].

Several of the published manuscripts attempted to correlate presence of CKD-A-RLS with age, gender, abnormalities of hemogram, iron, ferritin, serum lipids, electrolytes (particularly calcium and phosphorus) and parathyroid hormones. The results were inconsistent and controversial. Although also controversial, more studies have found predominance of women and increased incidence of sleep apnea, cerebrovascular accidents, depression and poor quality of life among patients affected by CKD-A-RLS. The correlation between CKD-A-RLS and hemodialysis parameters was also controversial, but CKD-A-RLS was observed in more patients with advanced kidney failure (beyond stage 3).

Two studies compared the prevalence of CKD-A-RLS in peritoneal dialysis with hemodialysis [34,35]. In both studies, the prevalence of CKD-A-RLS was substantially higher in peritoneal dialysis, 50% and 33% versus 23% in hemodialysis. In one study, using the cool dialysate improved the symptoms of RLS in CKD [36].

## Treatment of CKD-A-RLS

Limited studies have reported on the treatment of CKD-A-RLS; the data involves both non-pharmacological and pharmacological treatment. On non-pharmacological treatments, reports include the effect(s) of exercise, acupuncture, massage with different oils and infra-red light (Table 1).

**Table 1:** Non- pharmacological Treatments of CKD-A-RLS.


MODE OF TREATMENT	NUMBER OF PATIENTS	TYPE OF STUDY	DURATION OF TREATMENT	RESULTS	SIDE EFFECTS

Aerobic exercise [37]	Treatment 7 Control 7	Prospective, Open label	16 weeks	Reduced IRLS score by 42% Improved QoL: P = 0.03 Functional ability: P = 0.02 Sleep quality P = 0.01	None

Aerobic exercise [38]	Treatment 13 Control 13 All on hemodialysis	Prospective, Open label	16 weeks Bicycling 3 times/week	IRLS scores improved on week 16; no improvement of quality of life	None

Glycerin and lavender oil massage [39]	Glycerin 35 Lavender 35 Control 35 All on hemodialysis	Prospective Open label	45 minute, 3 times a week for one month	At the end of the study, both glycerin and lavender oil significantly improved symptoms (P < 0.05)	None

Lavender oil and sweet orange oil massage [40]	Lavender 35 Sweet orange 35 Control 35 All on hemodialysis	Double blind, Controlled	3 weeks, 3 times a week	At the end of the study, both glycerin and sweet orange significantly improved symptoms (P < 0.001)	None

Lavender oil massage [41]	Lavender 21 Control 21	Double blind Controlled	4 weeks	At the end of the study, lavender oil significantly improved symptoms (P < 0.0001)	None

Lavender oil massage [42]	Lavender 31 Control 26 (baby oil)	Placebo Controlled	10 minutes massage, 3 times per week for 4 weeks	RLS severity decreased and QoL improved significantly in the lavender group (P < 0.001)	None

Lavender oil massage [43]	Lavender 29 Control 30	Placebo Controlled	10 minutes, 3 times per week	RLS severity significantly decreased in the lavender group (P < 0.0001)	None

Application of infrared light at acuopoints [44]	Treatment 30 Control 30	Single blind Prospective	3 times per week for 3 weeks	Reduced IRLSRS scores but only during treatment	None


Pharmacological treatments consist mainly of treatment with dopaminergic drugs (levodopa, ropinirole), and pramipexole, as well as treatment with calcium channel blockers structurally similar to gabaaminobutiric acid (gabapentin and pregabalin) (Table 2).

**Table 2:** Pharmacological Treatment of CKD-A-RLS.


NAME OF THE DRUG (S)	DESIGN OF STUDY	NUMBER OF PATIENTS, DOSE	RESULTS	SIDE EFFECTS

Levodopa [45]	Double blind, cross over for 4 weeks	15, 100 & 200 mg	Improved leg movements, PLM index, sleep quality and QoL (Ps < 0.03)	Dry mouth and headaches

Gabapentin [46]	Double blind, crossover for 4 weeks (1 week wash out)	15, 200 & 300 mg	11 patients responded to gabapentin,1 to both placebo and gabapentin	Two drop-outs, one due to lethargy, one due to MI (unrelated to drug)

Rotigotine patch, Stage 2 CKD [47]	Single center Prospective, open label	14, 1 mg, 2 mg	Improved: severity of Symptoms (p < 0.003), QoL (P < 0.001, sleep (P < 0.001).	One patient:GI upset, no augmentation

Gabapentin [48]	Single center, Retrospective	59: GP 50 and 100 mg 125: controls	No effect	In gabapentin group, 17% discontinued treatment

Rotigotine patch [49] CKD/HD	Double blind, placebo controlled	15 Rotigotine 1 to 3 mg, 3 times daily for 3 weeks 10 Placebo	At the end of study: 10 of 15 and 2 of 10 showed significant improvement of RLS score in Rotigotine and placebo groups, respectively.	Nausea 20% Vomiting 15%

Low dose ropinirole vs aerobic exercise vs placebo 4 hours HD, 3 sessions/week [50]	Partially, double blind/placebo controlled	0.25 mg ropinirole, 2 h before sleep, 45 minutes cycling during each HD session Ropinirole 7 Exercise 15 Placebo 7	Ropinirole and aerobic exercise equally improved IRLS scores, QoL and depression. Ropinirole improved sleep quality	No side effects

Gabapentin (GP) versus Levodopa (LD) [51]	Double blind	GP: 42 LD: 40 GP: 200 mg/d LD: 110 mg/d Over 4 weeks	Both reduced IRLS scores but GP was more effective (P < 0.016). Both improved quality of sleep	Transient hypotension in two patients who took Levodopa. Increased day time sleepiness GP > LD

Gabapentin (GP) versus Levodopa (LD) [52] Hemodialysis: 3 times/week	Observational Cross sectional	GP: 14 LD: 12 GP: 200 mg/after each dialysis session LD: 110 mg/day 4 weeks treatment duration	IRLSS score was significantly improved after both Gabapentin and Levodopa treatment (P = 0.0001). Gabapentin was superior to L-Dopa in improving quality of sleep and QoL	Not mentioned

Gabapentin versus levodopa [53] Hemodialysis 3 times/week	Open label Prospective	GP# 15 LD#15 Gabapentin: 200 mg after each dialysis session LD: 125 mg, 2 hours before sleep	Both improved IRLSS scores. Gabapentin was superior to levodopa in improving sleep quality and latency, QoL (measured by SF36), general health and body pain	One patient dropped from the study due to gabapentin side effect (type not mentioned).

Ropinirole versus levodopa-SR [54] Chronic hemodialysis patients	Randomized, cross-over	10 Levodopa 190 mg/day Ropinirole 1.45 mg/day Duration 14 weeks (4 weeks each trial with 6 weeks washout)	At the end of study, ropinirole was superior to levodopa regarding improving scores of six item IRLS and increasing sleep time (P < 0.001) as well as improvement of clinical impression scorea (P < 0.01)	One patient taking levodopa withdrew from the study due to vomiting


GP: gabapentin; LD: levodopa; QOL-quality of life; HD: hemodialysis.

Additional therapeutic approaches for CKS-A-RLS include treatment with vitamins E and C which claimed alleviation of symptoms without side effects (Table 3).

**Table 3:** Vitamin C and E treatment for CKD-A-RLS.


TYPE OF VITAMIN	NUMBER OF PATIENTS	STUDY DESIGN	DOSE AND DURATION OF TREATMENT	RESULTS	SIDE EFFECTS

C and E [55]	60 Divided in 4 groups each including 15 patients Group 1: C + P Group 2: E + P Group 3: C + E Group 4: double placebo	Double blind-placebo controlled	Vitamin C: 200 mg/day Vitamin E: 400 mg/day All treatments for 8 weeks	At 8 weeks, patients treated with vitamin C or E or both demonstrated significant reduction of IRLS score compared to placebo. The difference between vitamin treated groups was not significant.	none

Intravenous Vitamin C [56]	90	Double blind placebo controlled	Intravenous Vitamin C, three times per week (given at the end of dialysis session for 8 weeks.	Quality of sleep and RLS scores improved significantly in patients who received vitamin C.	None

Compared Vitamin C with pramipexole [57]	45 Divided in three group each including 15 patients Group 1: Vitamin C Group 2: pramipexole Group 3-: placebo	Double blind placebo controlled	Vitamin C: 250 mg Pramipexole: 0.18 mg Vitamin C, pramipexole and placebo were given once daily for 8 weeks	Both Vitamin C and Pramipexole groups demonstrated significant improvement of IRLS scores after treatment (P < 0.001).	One patient in the pramipexole developed nausea and vomiting and excluded


Limited studies have been published on the therapeutic role of intravenous iron in dialysis patients with CKD-A-RLS (Table 4).

**Table 4:** Intravenous Iron Therapy for RLS-A-CKS.


TREATMENT	NUMBER OF PATIENTS	STUDY DESIGN	DOSE, DURATION	RESULTS	SIDE EFFECTS

Intravenous (IV) iron dextran [58]	25 Iron dextran: 11 Placebo: 14	Double blind- placebo controlled	Iron dextran: 1000 mg Study duration: 4 weeks. IRLS intensity scores were assessed weekly	At weeks 1 and 2 post-injection, patients in the Iron treated group showed significant reduction of IRLS scores (P = 0.01 and P = 0.03). At week 4, though still lower than placebo, the difference was not statistically significant	No difference in adverse effects between the two groups

Intravenous iron sucrose [59]	30 IV Iron Sucrose: 16 Placebo: 16	Randomized, placebo-controlled	Iron sucrose: 100 mg, three times/week for a total of 1000 mg. Study duration: 3 weeks	After two weeks, IRLS scores (compared to baseline) were significantly reduced compared to placebo group (P = 0.000). Improvement of IRLS score in the iron group continued for 4–24 weeks.	No adverse effects

Intravenous iron sucrose [60]	18 Iron sucrose:11 Placebo:7	Double blind- placebo controlled	500 mg of Iron sucrose was administered IV in two successive days for a total of 1000 mg Study duration: 4 weeks Primary outcome for RLS symptom improvement was global rating scale (GRS)	The trial was aborted half- way into the study since despite some improvement in GRS (at two weeks), authors predicted lack of robust response at the end of the study	Edema in either hands or feet (36%). Nausea or vomiting (36%). Hypotension (18%). dizziness (18%). abdominal pain (9%). All noted during infusion only.


GRS: Global rating Scale.

Among narcotic medications oxycodone (two blinded study [61,62]) (Table 5), and tramadol (two open label studies) have been reported to alleviate the symptoms of severe restless legs syndrome. No information on the use of oxycodone in CKD-A-RLS is available. Oxycodone needs to be used with caution in patients with kidney failure as the main mode of its elimination is renal [63].

**Table 5:** Double-blind, placebo-controlled studies of narcotic treatment in restless legs syndrome-.


AUTHORS AND DATE	NUMBER OF PATIENTS	DRUG, DOSE, STUDY DURATION	RESULTS	SIDE EFFECTS

*Walters et al, 1993 [61]	11	Oxycodone, 5 mg tablets, average dose 15.9 mg/day Study duration: two weeks	Patients rated improvement on the scale of 1–4: leg sensations, daytime sleepiness, motor restlessness and PLS. All Significantly improved (P < 0.5),	Mild constipation: two patients, mild lethargy: 1 patient

**Trenkwalder et al, 2009 [62]	267	Oxycodone/Naloxone (longacting), starting dose 5 mg/2.5 mg twice daily increasing up to 40/20 mg per research’s discretion. Study duration: 12 weeks	Mean International RLS Study group severity rating scale Sum score significantly improved in oxycodone group At week 12 (P < 0.0001)	Serious adverse effects: 3 in the double blind phase, 3 in the extension phase.(not specified).


* Double-blind, crossover; ** Double-blind, parallel design; PLS: periodic leg movements of sleep.

Six publications provided data on CKD-A- RLS in pediatric population with ages of 5 to 17 years [64,65,66,67,68,69]. In five of these studies [64,65,67,68,69], the prevalence of RLS was higher in CKD (15.3% to 35%) compared to normal population. All studies were open label. Three out of six were prospective. Information was taken in the clinic or via telephonic contact, often through the parents. In general, the symptoms were mild; only in two studies poor quality of sleep and impaired quality of life was mentioned. No other treatments were reported.

## Discussion

Our search found a higher incidence of RLS in chronic kidney disease compared to the normal population; for most studies, the reported incidence ranged between 15–30%. A few studies that reported a much higher incidence for RLS in CKD (51–84%) [13,14,15,16,17,18,19] represent distinct populations, mostly from middle eastern countries [13,14,15,16,17]. One of these studies had been published in 1995 [18], well before establishment of the modern diagnostic RLS criteria (Table 6).

**Table 6:** Diagnostic criteria defined for diagnosis of RLS by International Restless Legs Society, (last revision 2014).


1	An irresistible urge to move the legs, usually but not always accompanied by uncomfortable and unpleasant sensations in the legs

2	Symptoms that begin or worsen during the periods of inactivity, such as lying down or sitting

3	Symptoms are partially or totally relieved by movement

4	Symptoms only occur and are worse in the evening or night than during the day

5	The occurrence of the described features is not solely accounted for as symptoms primary to another medical or a behavioral condition (myalgia, venous stasis, leg edema, arthritis, leg cramps, positional discomfort, habitual foot tapping). The criteria published earlier in 2003 lacks the 5^th^ criteria.


The diversity of data found in this review is not surprising considering the fact that different investigators used different criteria for diagnosis of RLS (particularly before 2003). Furthermore, the studies represented findings in different stages of chronic kidney disease, hence not quite comparable. On the clinical sides, though the data is contradictory, considerably more studies reported positive correlations with increased mortality, increased cardiovascular complications, insomnia, and depression.

Non-pharmacological treatment, especially aerobic exercise and massage with lavender oil seems helpful in treatment of CKD-A-RLS. The Guideline Development Subcommittee of the American Academy of Neurology [70,71] recommends treating CKD-A-RLS with vitamin C and E based on one published class I study [55]. As a reducing agent, vitamin C plays an important role in iron metabolism. It increases absorption of iron from gastrointestinal tract and enhances the bioavailability of iron after intravenous iron injection. It also can mobilize iron from the reticuloendothelial system to transferrin [72,73].

Currently, dopaminergic medications (levodopa, ropinirole, pramipexole and rotigotine) and gabapentinoids (gabapentin and pregabalin) are recommended as the first line of drugs for treatment of essential RLS [74]. However, in case of CKD-A-RLS, more robust investigations for ropinirole and pramipexole are needed. Development of augmentation remains a worrisome issue with the use of dopaminergic drugs especially if long term therapy is contemplated. Currently, more practitioners prefer the use of direct dopamine agonists (ropinirole, pramipexole, rotigotine) over levodopa for treatment of CKD-A-RLS. One randomized controlled study has shown the superiority of ropinirole over levodopa for treatment of CKD-A-RLS (Table 2) [54]. The same preference applies to gabapentin over levodopa based on three small comparative studies (Table 2).

Due to renal clearance, dose adjustment is necessary when oral dopaminergic drugs or gabapentinoid medications (gabapentin and pregabalin) are going to be used for treatment of CKD-A-RLS. In case of gabapentin and pregabalin the following dosing schedule is recommended by Chincholkar et al [75] (Table 7).

**Table 7:** Dose adjustment recommended for gabapentin and pregabalin in kidney failure [66].


DRUG’S NAME	BASED ON CREATINE CLEARANCE (ML/MINUTE)	RECOMMENDED DOSE GIVEN IN THREE DIVIDED DOSES IN ALTERNATE DAYS

Gabapentin	50–79	600–180 mg

	30–49	300–900 mg

	15–29	150–600

	<15	150–300

Pregabalin	Based on eGFR, mL/min/1.73 m^2^	

	30–60	Initially 75 mg, maximum 300 mg daily

	15–30	Initially 25–50 mg, maximum 300 mg in two divided doses

	<30	Initially 25 mg once daily and maximum 75 mg once daily


Ropinirole was approved by FDA for treatment of restless legs syndrome in 2005 and pramipexole in 2006. In an open label study of 10 patients affected by advanced kidney disease and on dialysis, Miranda et al [76] reported a significant improvement of RLS severity scores (using the criteria set by International RLS Study Group) after treatment with pramipexole (mean dose of 0.25 mg/day). The mean time of follow up was 8 months. Currently, two randomized, double- blind studies are ongoing with aims of assessing the efficacy of ropinirole and pramipexole in CKD-A-RLS [77,78], the results of which will hopefully, be available soon.

Rotigotine as a skin patch has the advantage of bypassing drug absorption through the GI tract which is often affected in patients with chronic kidney disease. Renal clearance of rotigotine is also not influenced by kidney disease. Even in advanced kidney failure the level of unconjugated rotigotine does not change indicating no need for dose adjustment [79]. For these advantages, treatment with rotigotine deserves further investigation. Intravenous iron using iron dextran and iron sucrose have been helpful in reducing intensity of RLS in chronic kidney disease especially in case of iron deficiency [58,59,60].

Up to 65% of patients with CKD in clinical examination demonstrate evidence of peripheral Neuropathy [80]. Peritoneal and hemodialysis can improve mild peripheral neuropathy but their effect on severe peripheral neuropathy is not adequately studied [81]. A clinical trial with two years of follow up demonstrated failure of renal transplantation to improve CKD associated peripheral neuropathy [82].

## Conclusion

This updated review showed that restless legs syndrome is two to three times more common in chronic kidney disease compared to the general population. When assessed, more patients with CKD-A-RLS demonstrated increased mortality, increased incidence of cardiovascular accident, depression, insomnia and impaired quality of life compared to CKD patients without RLS. Dopaminergic drugs such as levodopa, ropinirole, pramipexole and rotigotine as well as calcium channel blockers (gabapentin and pregabalin) are helpful for treatment of RLS. High quality studies with these agents are currently underway and hopefully confirm the efficacy and practicality of using these drugs in CKD-A-RLS. Treatment with Vitamin C and E is recommended for CKD-A-RLS. Some studies have shown that aerobic exercise and massage with lavender oil can improve symptoms of CKD-A- RLS suggesting that these measures can be useful as adjunct therapy.
